# Gestational weight gain standards based on women enrolled in the Fetal Growth Longitudinal Study of the INTERGROWTH-21^st^ Project: a prospective longitudinal cohort study

**DOI:** 10.1136/bmj.i555

**Published:** 2016-02-29

**Authors:** Leila Cheikh Ismail, Deborah C Bishop, Ruyan Pang, Eric O Ohuma, Gilberto Kac, Barbara Abrams, Kathleen Rasmussen, Fernando C Barros, Jane E Hirst, Ann Lambert, Aris T Papageorghiou, William Stones, Yasmin A Jaffer, Douglas G Altman, J Alison Noble, Maria Rosa Giolito, Michael G Gravett, Manorama Purwar, Stephen H Kennedy, Zulfiqar A Bhutta, José Villar

**Affiliations:** 1 Nuffield Department of Obstetrics & Gynaecology and Oxford Maternal & Perinatal Health Institute, Green Templeton College, University of Oxford, Oxford, UK; 2 School of Public Health, Peking University, Beijing, China; 3 Centre for Statistics in Medicine, Botnar Research Centre, University of Oxford, Oxford, UK; 4 Universidade Federal do Rio de Janeiro/Rio de Janeiro Federal University, Instituto de Nutrição Josué de Castro/Nutrition Institute, Departamento de Nutrição Social e Aplicada, Rio de Janeiro, Brazil; 5 School of Public Health, University of California, Berkeley, CA, USA; 6 Division of Nutritional Sciences, Cornell University, Ithaca, NY, USA; 7 Programa de Pós-Graduação em Epidemiologia, Universidade Federal de Pelotas, Pelotas, RS, Brazil; 8 Programa de Pós-Graduação em Saúde e Comportamento, Universidade Católica de Pelotas, Pelotas, RS, Brazil; 9 Faculty of Health Sciences, Aga Khan University, Nairobi, Kenya; 10 School of Medicine, University of St Andrews, St Andrews, Scotland, UK; 11 Department of Family and Community Health, Ministry of Health, Muscat, Sultanate of Oman; 12 Department of Engineering Science, University of Oxford, Oxford, UK; 13 Direttore SC consultori familiari e pediatria di comunità, Torino, Italy; 14 Global Alliance to Prevent Prematurity and Stillbirth (GAPPS), Seattle Children’s, Seattle, WA, USA.; 15 Nagpur INTERGROWTH-21^st^ Research Centre, Ketkar Hospital, Nagpur, India; 16 Centre of Excellence in Women and Child Health, Aga Khan University, Karachi, Pakistan; 17 Centre for Global Child Health, Hospital for Sick Children, TN, Canada

## Abstract

**Objective** To describe patterns in maternal gestational weight gain (GWG) in healthy pregnancies with good maternal and perinatal outcomes.

**Design** Prospective longitudinal observational study.

**Setting** Eight geographically diverse urban regions in Brazil, China, India, Italy, Kenya, Oman, United Kingdom, and United States, April 2009 to March 2014.

**Participants** Healthy, well nourished, and educated women enrolled in the Fetal Growth Longitudinal Study component of the INTERGROWTH-21^st ^Project, who had a body mass index (BMI) of 18.50-24.99 in the first trimester of pregnancy.

**Main outcome measures** Maternal weight measured with standardised methods and identical equipment every five weeks (plus/minus one week) from the first antenatal visit (<14 weeks’ gestation) to delivery. After confirmation that data from the study sites could be pooled, a multilevel, linear regression analysis accounting for repeated measures, adjusted for gestational age, was applied to produce the GWG values.

**Results** 13 108 pregnant women at <14 weeks’ gestation were screened, and 4607 met the eligibility criteria, provided consent, and were enrolled. The variance within sites (59.6%) was six times higher than the variance between sites (9.6%). The mean GWGs were 1.64 kg, 2.86 kg, 2.86 kg, 2.59 kg, and 2.56 kg for the gestational age windows 14-18^+6^ weeks, 19-23^+6^ weeks, 24-28^+6^ weeks, 29-33^+6^ weeks, and 34-40^+0^ weeks, respectively. Total mean weight gain at 40 weeks’ gestation was 13.7 (SD 4.5) kg for 3097 eligible women with a normal BMI in the first trimester. Of all the weight measurements, 71.7% (10 639/14 846) and 94.9% (14 085/14 846) fell within the expected 1 SD and 2 SD thresholds, respectively. Data were used to determine fitted 3rd, 10th, 25th, 50th, 75th, 90th, and 97th smoothed GWG centiles by exact week of gestation, with equations for the mean and standard deviation to calculate any desired centiles according to gestational age in exact weeks.

**Conclusions** Weight gain in pregnancy is similar across the eight populations studied. Therefore, the standards generated in this study of healthy, well nourished women may be used to guide recommendations on optimal gestational weight gain worldwide.

## Introduction

Associations between insufficient or excessive gestational weight gain (GWG) and short and long term maternal and child health outcomes are well described.^1^ Insufficient weight gain has been linked with increased risks of low birth weight, small for gestational age, and preterm birth, while excessive gain has been associated with large for gestational age, gestational diabetes, preterm birth, caesarean section, infant mortality, postpartum weight retention, and childhood obesity.^2^ ^3^ ^4^ ^5^ ^6^ ^7^ ^8^ ^9^ Pregnant women are therefore routinely weighed in clinical settings. The benefits of doing so, however, are debatable in the absence of appropriate guidelines or even agreement on what constitutes adequate weight gain.^10^ ^11^

In 1970, the Institute of Medicine/National Research Council reviewed the available evidence on GWG that resulted in good pregnancy outcomes, with subsequent revisions in 1990 and 2009.^1^ ^12^ ^13^ The latest guidelines evaluated the trade offs between maternal and child health outcomes and weight gain during pregnancy, including the risks of small for gestational age and preterm birth with inadequate GWG and the increased rates of caesarean section and postpartum weight retention with excessive GWG. Based on a recent systematic review, however, these guidelines were all derived from country specific studies that varied in sample selection, study design, and methods of data collection and statistical analysis.^14^ In the United Kingdom, “routine weighing during pregnancy should be confined to circumstances in which clinical management is likely to be influenced.”^15^ In countries where routine weighing is recommended, most current guidelines are based on relating observed GWG to pregnancy outcomes and then determining the range of weight gain with the lowest perinatal risk,^1^ ^16^ ^17^ ^18^ although other authors have attempted to select populations with good perinatal outcomes and then retrospectively determine the associated GWG range.^19^ ^20^ ^21^ ^22^

The World Health Organization recommends that a reference for GWG be based on prospective longitudinal studies of selected populations with a low incidence of maternal and fetal complications, where anthropometric measures are collected before and during pregnancy and postpartum.^23^ The same “prescriptive” approach was adopted by WHO in producing international growth standards for children aged 0-5 years that have now been adopted by more than 125 countries worldwide,^24^ and by the International Fetal and Newborn Growth (INTERGROWTH-21^st^) Consortium for the 21^st ^Century in producing standards for early pregnancy dating,^25^ fetal growth,^26^ newborn size,^27^ and postnatal growth for preterm infants.^28^ We examined data on GWG obtained, according to WHO recommendations, from healthy pregnant women who were free from identifiable major medical, nutritional, or social and major environmental risk factors.^26^ ^29^ ^30^ The women had pregnancies with good maternal and perinatal outcomes.^31^ Based on these data, we report GWG patterns from normal weight women.

## Methods

### Study site and population selection

INTERGROWTH-21^st^ was a multicentre multiethnic population based project conducted between April 2009 and March 2014 in eight well defined urban sites: Pelotas (Brazil), Turin (Italy), Muscat (Oman), Oxford (UK), Seattle (US), Shunyi County in Beijing (China), the central area of Nagpur (India), and the Parklands suburb of Nairobi (Kenya). The primary aim was to produce international standards for fetal, newborn, and preterm growth using the same conceptual framework as the WHO Multicentre Growth Reference Study^24^ ^30^ ^32^ to complement the existing WHO Child Growth Standards.

We recruited women who started antenatal care before 14 weeks’ gestation with reliable menstrual dates and a confirmatory ultrasound dating scan who met the entry criteria of optimal health, nutrition, education, and socioeconomic status and were not exposed during pregnancy to environmental hazards.^25^ ^29^ ^30^ These low risk women constituted the population of the Fetal Growth Longitudinal Study (FGLS) component of the INTERGROWTH-21^st^ Project.^26^

### Measurements

A detailed manual with instructions for all adult measurement techniques, the methods for multicentre standardisation of those measures, and the procedures for the calibration and maintenance of equipment have been published elsewhere.^33^ ^34^ ^35^ All documentation, protocols, data collection forms, and electronic transfer strategies are available at www.intergrowth21.org. Briefly, the women’s height and weight were measured in duplicate with a Seca 264 stadiometer and Seca 877 scale (Seca, Germany), respectively, on study entry between 9 and 13^+6^ weeks’ gestation. A first trimester body mass index (BMI) was calculated and categorised as normal weight (18.50-24.99) or overweight (25.00-29.99), according to the WHO definition.^36^ The same standardised methods and clinical procedures were used to measure maternal weight every five weeks (plus/minus one week) until delivery, so that the possible ranges after recruitment in which weight was measured were 14-18, 19-23, 24-28, 29-33, 34-38, and 39-42 weeks’ gestation.^35^

### Statistical analyses

GWG was calculated as the measured weight at each antenatal visit minus the measured weight in the first trimester. According to prespecified criteria, we excluded pregnancies complicated by fetal death or congenital abnormality, catastrophic or severe medical conditions (such as cancer or HIV), those with severe unanticipated conditions related to pregnancy that required admission to hospital (such as eclampsia or severe pre-eclampsia), and those identified during the study who no longer fulfilled the entry criteria (such as women who started smoking during pregnancy or had an episode of malaria).

The first step was to assess variation in GWG across sites and whether we could pool the data. A detailed analysis of the methods used to assess the similarity of fetal and newborn data from all eight INTERGROWTH-21^st^ sites to permit pooling has been reported elsewhere.^31^ ^37^ We applied the same methods to the GWG data by using variance component analysis (analysis of variance (ANOVA)) to calculate the percentage of variance in the longitudinal weight measurements from variance between sites adjusted for gestational age (fixed effects) while sites and individuals were treated as random effects, and a standardised site difference (SSD), similar to a z score, calculated as the difference between the mean of one site and the mean of all sites together. Each difference was then expressed as a proportion of the all sites’ standard deviation (SD) (that is, SD of the data pooled across all sites) at each corresponding gestational age. The SSD allows for direct comparisons across gestational age windows, and we prespecified a value of ≤0.5 as adequate for combining data from all sites. This is similar to the cut off used in the WHO Multicentre Growth Reference Study to create international standards for infant and child growth.^38^

In a second step we constructed smoothed centiles of GWG according to gestational age. The statistical methods we used were informed by the recommendations of Altman and Royston^39^ ^40^ and recent literature reviews.^41^ ^42^

We applied a multilevel linear regression analysis accounting for repeated measures, adjusting for gestational age, which we treated as a fixed effect, whereas sites and individuals were treated as random effects.^38^ As weight gain exhibited a non-normal distribution, we log transformed (natural log) data to stabilise variance and transform the data to normality. We added a constant 8.5 for normal weight women to all values to shift the minimum value of the distribution to 1 to ensure no negative values when we modelled on the log scale. The best fitting powers for the mean weight gain were provided by second degree fractional polynomials and further modelled in a multilevel framework to account for the longitudinal design of the study (repeated measures). The data structure comprises two levels—that is, measurements within and between women. Therefore, we fitted a random effects model (two level hierarchical structure) to the longitudinal GWG measurements as a function of gestational age using the *runmlwin* package in STATA.^43^ To obtain an equation for the SD, we modelled the resulting variance components from the multilevel model that accounts for the correlations between and within women using fractional polynomials. The SD was modelled on the log scale to stabilise variance. Assessment of goodness of fit incorporated a visual inspection of the overall model fit by comparing empirical centiles (calculated per completed week of gestation—for example, 38 weeks’ gestation=38-38^+6^ weeks’ gestation) to the fitted centiles; a quantile-quantile (q-q) plot of the residuals; and a plot of fitted z scores across gestational ages.

As the first weight measurement was taken between 9 and 13^+6^ weeks’ gestation, we performed a sensitivity analysis to explore the likelihood of potential bias that might arise as a result of this classification. Based on a reported range in weight gain of 0.5-2 kg in the first trimester,^1^ we performed a post hoc analysis to estimate the proportion of women who were within 2 kg of the lower limit in the normal weight group (and so could have been underweight before conception) and, similarly, those within 2 kg of the lower cut off for overweight women, as they might actually have been normal weight before conception. The data were modelled with the same analytical strategy and the resultant centiles compared with those obtained from our original classification of normal weight (that is, based on the first trimester BMI). All analyses were performed in STATA, version 11.2, software (StataCorp LP, College Station, TX, US). Furthermore, to rule out potential bias from caesarean section, we performed a sensitivity analysis excluding all births by caesarean section and refitting the final model to the remaining data and compared this with the model using all the data.

### Patient involvement

No patients were involved in setting the research question or the outcome measures, nor were they involved in the design and implementation of the study. There are no plans to involve patients in dissemination.

## Results

A total of 13 108 pregnant women at <14 weeks’ gestation were screened (fig 1), and 4607 met the eligibility criteria, provided consent, and were enrolled in the Fetal Growth Longitudinal Study. The contribution from each site to the total enrolled sample population ranged from 311 (7%) for the US to 640 (14%) for the UK.

**Figure f1:**
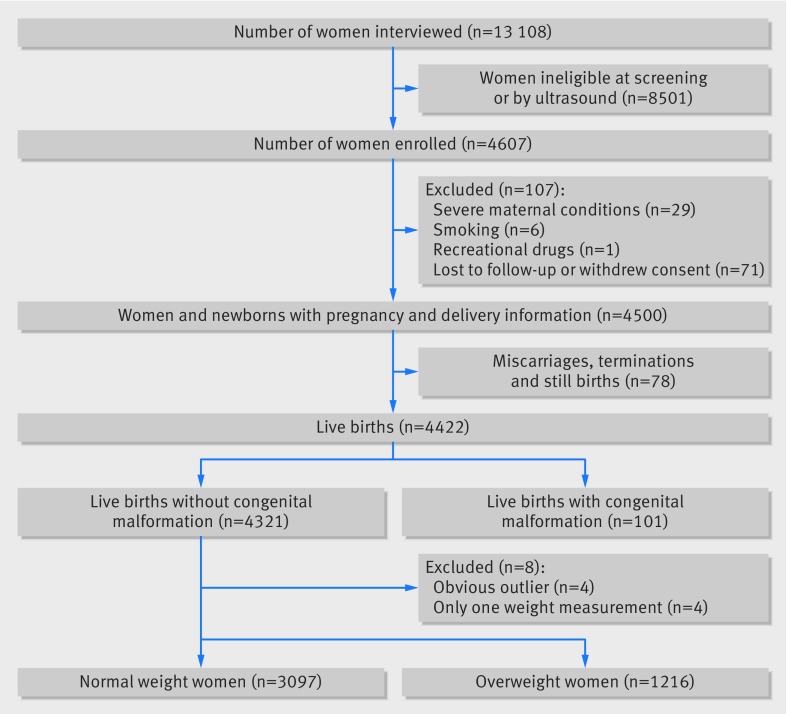
**Fig 1** Flowchart for selecting women included in study of gestational weight gain

The most common reasons for ineligibility were maternal height <153 cm (1022/8501; 12%), BMI ≥30 (1009/8501; 12%), and age <18 or >35 years (915/8501; 11%) at screening. During the pregnancy, 71 women were lost to follow-up or withdrew consent and 36 were excluded (during pregnancy 29 had severe medical conditions, six took up smoking, and one used recreational drugs). After exclusion of 78 miscarriages, terminations, or stillbirths, there were 4422 live singleton births of which a further 101 were excluded because of congenital malformations. Of the 4321 remaining women, eight were excluded from the analysis (four with only one weight measurement during pregnancy and four who were obvious outliers because of illogical values that could not be corrected during data cleaning). We excluded nine observations with extreme weight changes (defined as a gain or loss of >5 kg/week).

Our final sample therefore consisted of 4313 women who contributed 24 977 weight measurements. Of these, 3097 (72%) women had normal weight in the first trimester. Here we report the analyses pertaining to these normal weight women whose data were used to construct the international GWG standard.

The demographic characteristics of the study cohort were similar across the eight sites and have been reported elsewhere.^31^ Women had a median of six weight measurements (range 2-7); median gestational age at first antenatal visit was 11.9 weeks (SD 1.4 weeks); mean maternal age was 28.2 (SD 3.8) years; 97% (3020/3097) were married or living with a partner, and 72% (2230/3097) were nulliparous. Table 1 shows sociodemographic information and pregnancy and perinatal events. Fig 2 shows an example of the crude weight gain trajectories of a simple random sample of 100 normal weight women, illustrating the longitudinal design of the study.

**Table 1 tbl1:** Baseline characteristics and pregnancy outcomes of the normal-weight women. Values are mean (SD) for continuous variables, and number (percentage) for categorical variables

	Normal BMI (n=3097)
Parents	
Maternal age (years) (SD)	28.2 (3.8)
Maternal height (cm) (SD)	162.3 (5.9)
Maternal weight (kg) (SD)	57.2 (6.5)
Paternal height (cm) (SD)	174.2 (7.3)
Body mass index (SD)	21.7 (1.8)
Gestational age at first visit (weeks) (SD)	11.9 (1.4)
Years of formal education (years) (SD)	15.1 (2.9)
Haemoglobin level before 15 weeks’ gestation (g/dL) (SD)	12.5 (1.1)
Married/cohabiting (%)	3020 (97.3)
Nulliparous (%)	2230 (71.8)
Pre-eclampsia (%)	12 (0.4)
Pyelonephritis (%)	9 (0.3)
Any sexually transmitted infection (%)	1 (0.0)
Spontaneous initiation of labour (%)	2127 (68.5)
PPROM (<37 weeks’ gestation) (%)	46 (1.5)
Caesarean section (%)	1036 (33.4)
Mother admitted to intensive care unit (%)	9 (0.3)
Infants	
NICU admission >1 day (%)	160 (5.2)
Preterm (<37 weeks’ gestation) (%)	125 (4.0)
Preterm and spontaneous onset of labour (%)	82 (2.6)
Term LBW (<2500 g; ≥37 weeks) (%)	99 (3.2)
Birth weight >4.0 kg (%)	144 (4.7)
Neonatal mortality (%)	4 (0.1)
Male sex (%)	1534 (49.4)
Exclusive breastfeeding at discharge (%)	2698 (86.9)
Mean (SD) weight (kg) (≥37 weeks’ gestation)	3.2 (0.4)
Mean (SD) length (cm) (≥37 weeks’ gestation)	49.3 (1.9)
Mean (SD) head circumference (cm) (≥37 weeks’ gestation)	33.8 (1.3)

PPROM=preterm pre-labour rupture of membranes; NICU=neonatal intensive care unit; LBW=low birthweight.

**Figure f2:**
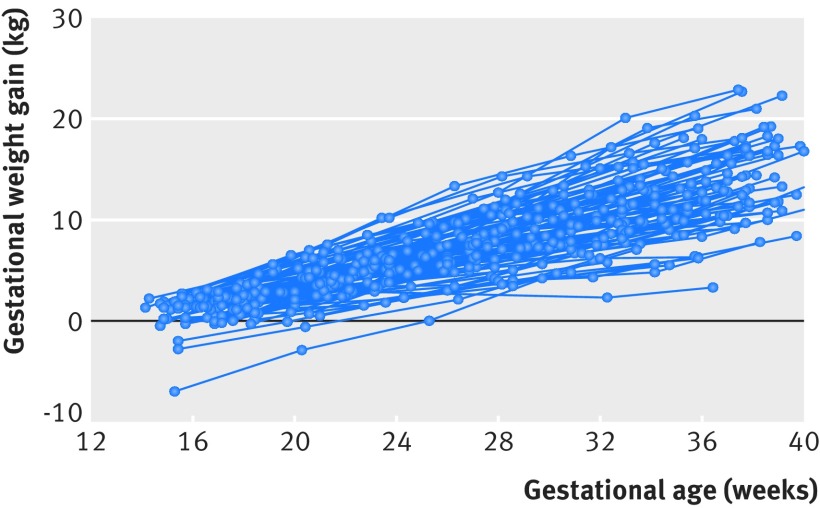
**Fig 2** Trajectories of gestational weight gain of 100 randomly selected normal weight healthy women with uncomplicated live singleton births

We explored the variation in GWG among the sites; the variance within sites (59.6%) was six times higher than the variance between sites (9.6%). The all sites’ SD for GWG ranged from 1.45 kg at 14-19^+6^ weeks’ gestation to 1.61 kg at 34-40 weeks’ gestation. Within five gestational age windows from 14 weeks to 40^+0^ weeks, representing 40 comparisons, 37 had standardised site differences (SSDs) ≤0.5 (as prespecified in the protocol) of the SD of all sites combined (fig 3, table 2). The three comparisons that were higher than 0.5 SSD were from China, but the difference was <0.5 at 14-18^+6^ weeks’ gestation and at 34-40 weeks’ gestation (0.34 and 0.21, respectively).

**Figure f3:**
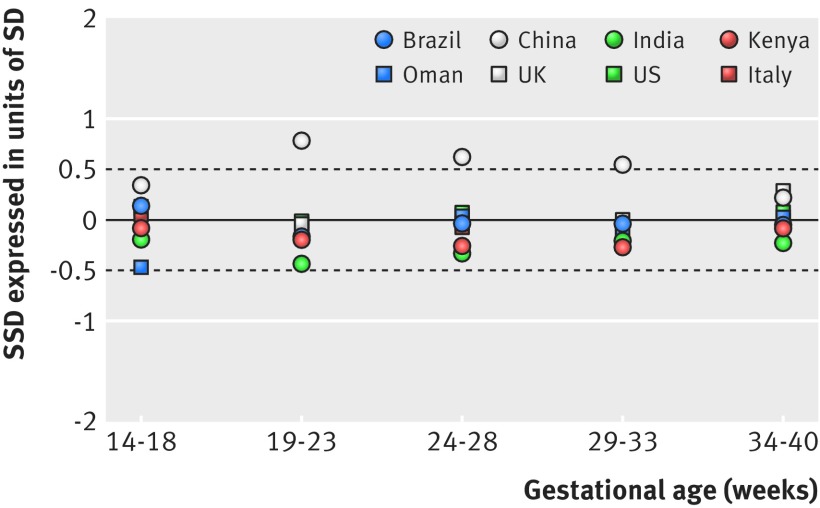
**Fig 3** Standardised site difference (SSD) for gestational weight gain in the Fetal Growth Longitudinal Study of the INTERGROWTH-21^st^ Project. SSD calculated by (site mean of gestational weight gain at each gestational age interval minus all sites mean of gestational weight gain at the same gestational age interval)/all sites’ SD of gestational weight gain at the same gestational age interval. SSD was adjusted at median gestational age for all sites at each gestational age interval

**Table 2 tbl2:** All sites and individual site means (SD) for gestational weight gain (kg) of normal weight women

Gestational age and Country	No of measures	Adjusted mean (SD) GWG (kg)	Standardised site difference (SSD)*
14-18^+6^ weeks			
Brazil	252	1.84 (1.34)	0.14
China	476	2.14 (1.28)	0.34
India	441	1.36 (1.30)	−0.20
Kenya	355	1.52 (1.65)	−0.09
Oman	360	0.96 (1.53)	−0.47
UK	408	1.83 (1.44)	0.13
USA	221	1.79 (1.40)	0.10
Italy	392	1.71 (1.29)	0.05
All	2905	1.64 (1.45)	0.00
19-23^+6^ weeks			
Brazil	252	2.62 (1.11)	−0.16
China	485	4.00 (1.57)	0.79
India	448	2.22 (1.33)	−0.43
Kenya	356	2.57 (1.46)	−0.20
Oman	366	2.73 (1.31)	−0.08
UK	413	2.80 (1.26)	−0.04
USA	211	2.84 (1.40)	−0.01
Italy	384	2.75 (1.24)	−0.07
All	2915	2.86 (1.46)	0.00
24-28^+6^ weeks			
Brazil	251	2.81 (1.46)	−0.04
China	448	3.77 (1.68)	0.62
India	455	2.37 (1.40)	−0.34
Kenya	360	2.48 (1.29)	−0.26
Oman	370	2.91 (1.39)	0.03
UK	412	2.81 (1.27)	−0.04
USA	204	2.97 (1.30)	0.07
Italy	375	2.75 (1.35)	−0.08
All	2875	2.86 (1.47)	0.00
29-33^+6^ weeks			
Brazil	261	2.53 (1.43)	−0.04
China	545	3.41 (1.61)	0.55
India	428	2.28 (1.48)	−0.21
Kenya	355	2.18 (1.39)	−0.27
Oman	363	2.43 (1.39)	−0.11
UK	417	2.59 (1.23)	0.00
USA	216	2.52 (1.73)	−0.04
Italy	373	2.37 (1.35)	−0.15
All	2958	2.59 (1.51)	0.00
34-40^+0^ weeks			
Brazil	269	2.48 (1.57)	−0.05
China	485	2.91 (1.40)	0.22
India	406	2.19 (1.30)	−0.23
Kenya	388	2.42 (2.41)	−0.09
Oman	406	2.60 (1.36)	0.02
UK	534	2.61 (1.26)	0.03
USA	220	2.68 (2.14)	0.07
Italy	370	2.55 (1.40)	−0.01
All	3078	2.56 (1.61)	0.00

*Calculated by: (site mean of GWG−all sites mean of GWG at each gestational age interval)/all sites’ SD of GWG. SSD adjusted at median gestational age for all sites at each gestational age interval with estimates from final regression model.

The mean GWGs were 1.64 kg, 2.86 kg, 2.86 kg, 2.59 kg, and 2.56 kg for the gestational age windows 14-18^+6^ weeks, 19-23^+6^ weeks, 24-28^+6^ weeks, 29-33^+6^ weeks, and 34-40^+0^ weeks, respectively (table 2). Of all the weight measurements, 71.7% (10 639/14 846) and 94.9% (14 085/14 846) fell within the expected 1 SD and 2 SD thresholds, respectively, which compares well with 68% and 95% theoretically expected under normality assumptions. On average, across all gestational ages, the absolute magnitude of differences between the observed (empirical) and smoothed centiles was 0.18 kg for the median, 0.37 kg for the 3rd centile, and 0.06 kg for the 97th centile (fig 4).

**Figure f4:**
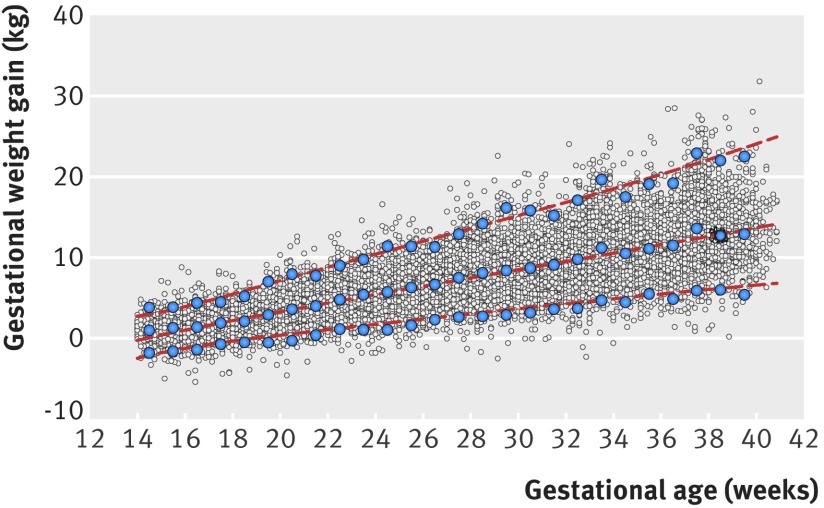
**Fig 4** Fitted 3rd (bottom dashed line), 50th (middle dashed line), and 97th (top dashed line) smoothed centile curves for gestational weight gain among normal weight women. Large blue circles show empirical values for each week of gestation, and small grey circles show actual observations

Table 3 provides the values of the smoothed week specific GWG according to gestational age of selected centiles (that is, 3rd, 10th, 25th, 50th, 75th, 90th, and 97th), which are shown graphically in figure 5. We have also provided the corresponding equations for the mean and SD from the multilevel regression model (table 4), allowing for calculation of any desired centiles according to gestational age in exact weeks. For example, centiles can be calculated as mean ±z×SD, where z is −1.88, −1.645, −1.28, 0, 1.28, 1.645, and 1.88 for the 3rd, 5th, 10th, 50th, 90th, 95th, and 97th centiles, respectively.

**Table 3 tbl3:** Smoothed centiles for gestational weight gain (GWG) for women of normal weight (BMI 18.50-24.99) according to gestational age

Gestational age (weeks)	No of measures	Centiles for GWG (kg)						
		3rd	10th	25th	50th	75th	90th	97th
14	260	−2.34	−1.73	−1.07	−0.25	0.65	1.54	2.50
15	473	−1.77	−1.14	−0.45	0.39	1.32	2.24	3.23
16	705	−1.26	−0.60	0.13	1.01	1.99	2.95	3.98
17	851	−0.80	−0.09	0.67	1.61	2.64	3.65	4.75
18	639	−0.37	0.38	1.19	2.19	3.29	4.36	5.53
19	324	0.03	0.82	1.69	2.75	3.92	5.07	6.31
20	532	0.41	1.25	2.17	3.30	4.55	5.78	7.11
21	627	0.77	1.66	2.64	3.84	5.17	6.49	7.91
22	715	1.11	2.05	3.10	4.37	5.79	7.19	8.72
23	717	1.45	2.44	3.54	4.90	6.41	7.90	9.52
24	399	1.77	2.82	3.98	5.42	7.02	8.61	10.34
25	500	2.09	3.19	4.42	5.94	7.63	9.31	11.15
26	599	2.40	3.56	4.85	6.45	8.24	10.02	11.97
27	675	2.71	3.93	5.28	6.96	8.85	10.73	12.79
28	702	3.02	4.29	5.71	7.47	9.45	11.43	13.61
29	493	3.33	4.65	6.14	7.98	10.06	12.14	14.44
30	526	3.63	5.01	6.56	8.49	10.67	12.86	15.27
31	533	3.94	5.37	6.99	9.00	11.28	13.57	16.10
32	691	4.24	5.73	7.41	9.52	11.89	14.29	16.94
33	715	4.55	6.10	7.84	10.03	12.51	15.01	17.78
34	498	4.85	6.46	8.27	10.55	13.12	15.73	18.62
35	533	5.16	6.82	8.70	11.06	13.74	16.46	19.47
36	514	5.47	7.19	9.14	11.58	14.37	17.19	20.32
37	858	5.78	7.56	9.57	12.11	14.99	17.92	21.18
38	402	6.10	7.93	10.01	12.63	15.62	18.66	22.04
39	230	6.41	8.30	10.45	13.16	16.25	19.40	22.91
40	82	6.73	8.68	10.89	13.69	16.89	20.15	23.79
Total No of measures	14 793	—	—	—	—	—	—	—

**Figure f5:**
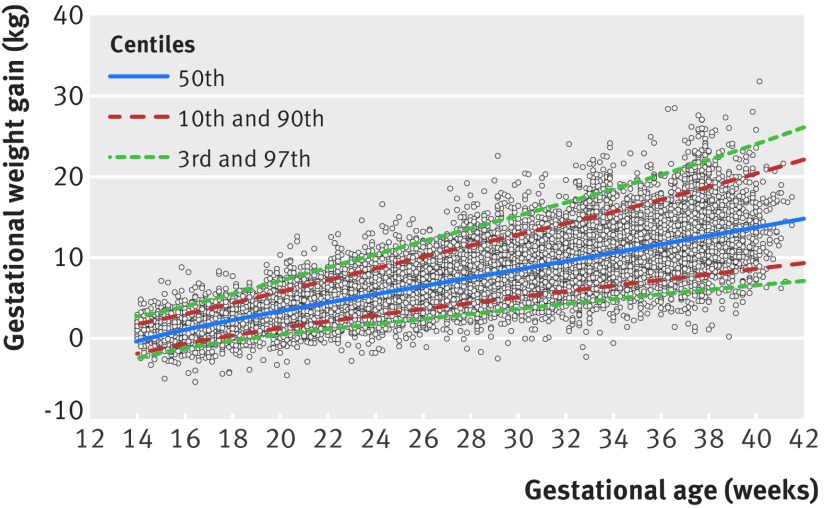
**Fig 5** Smoothed centile curves at 3rd, 10th, 50th, 90th, and 97th centiles for gestational weight gain among healthy normal weight women with uncomplicated live singleton births

**Table 4 tbl4:** Equations for estimating mean and standard deviation (SD) of gestational weight gain in normal weight women according to exact gestational age (weeks)*

Estimate	Regression equation
Mean; log (GWG)	1.382972−56.14743×GA^−2^+0.2787683×GA^0.5^
SD; log (GWG)	0.2501993731+142.4297879×GA^−2^−61.45345×GA^−2^×LN(GA)

GA=exact gestational age in weeks.

*All logarithms are natural logarithms. Using equations of mean and SD one can easily compute any desired centiles using relation *P*^th^ centile=mean+*K*SD where *K* is normal equivalent deviate (z score) corresponding to particular centile—for example, *K*=1.88 for 97th centile and −1.88 for 3rd centile, and SDs in this equation are predicted estimates from the regression analysis. For example to calculate gestational weight gain at 34 weeks;

C50 for GWG=exp((1.382972−56.14743×34^−2^+0.2787683×34^0.5^)+(0×(0.2501993731+142.4297879×34^−2^ − 61.45345*34^−2^*LN(34)))) – 8.75

C3 for GWG=exp((1.382972−56.14743×34^−2^+0.2787683×34^0.5^)+(−1.88×(0.2501993731+142.4297879×34^−2^ − 61.45345*34^−2^×LN(34)))) – 8.75

C97 for GWG=exp((1.382972− 56.14743×34^−2^+0.2787683×34^0.5^)+(1.88×(0.2501993731+142.4297879×34^−2^ −61.45345*34^−2^×LN(34)))) – 8.75

The sensitivity analyses performed to assess the impact of potential misclassification of BMI status resulted in 30% (932/3097) of women being excluded from the normal weight group and 53% (n=639/1216) of women being added from the overweight group to form a reclassified group of normal weight women (n=2804 women, 17 579 observations). The resultant centile values were remarkably similar and indistinguishable when we superimposed them on the normal weight GWG chart. Sensitivity results when we excluded women with caesarean sections had minimal effect compared with results using all the data (data not shown).

Tables containing the mean and SD, centile values, and z scores by gestational age, expressed in completed weeks’ gestation (as recommended by WHO ICD10^44^), and printable charts are available at www.intergrowth21.org.

## Discussion

### Principal findings

Despite the range of cultures, behaviours, clinical practices, and traditions, which can strongly influence gestational weight gain (GWG), we observed strikingly similar patterns of weight gain in the populations studied, reflecting their overall good health and living conditions, nutritional status, and access to adequate standardised healthcare. The proportion of total variance explained by population differences was <10% of the total variance. This finding indicates not only that separate GWG charts for women from different ethnic/racial groups are not required, as is the case for growth standards from early pregnancy to 5 years of age,^24^ ^26^ ^27^ but that the observed differences by race/ethnicity reported in some studies^45^ ^46^ ^47^ ^48^ are more likely caused by socioeconomic, medical, cultural, and nutritional factors than true biological differences in the process of nutrient absorption or fat deposition among healthy women. We adopted a prescriptive approach, employed highly trained anthropometrists to measure maternal weight prospectively in duplicate, and used uniform and standardised measurement equipment and protocols. We used the patterns in weight gain in women with a normal BMI in early pregnancy to produce international standards, using statistical techniques that account for repeated measurements within women at one site and between women across sites. We developed a standard, as well as the accompanying centile chart and simple formulae, to allow any desired centiles or z scores to be calculated. These tools complement the already published fetal growth, neonatal size, and postnatal growth of preterm infant standards from the INTERGROWTH-21^st^ Project.^26^ ^27^ ^28^

### Comparison with other studies

Comparisons with previous studies on this subject are difficult because of wide variations in study designs, methods, and populations selected. In particular, some studies based GWG on maternally recalled weight before pregnancy, while we measured weight using standardised methods at the first trimester visit. Nonetheless, the weight gain at term of women in the Fetal Growth Longitudinal Study (13.7 kg) was comparable with the range recommended in 2009 by the Institute of Medicine/National Research Council for normal weight women (11.5-16.0 kg) and optimal GWG reported for a multiethnic Singaporean population (13.7 kg), but about 2-3 kg less than that for low risk urban populations in Leuven, Belgium (15.9 kg) and Pittsburgh, USA (16.4 kg).^1^ ^16^ ^21^ ^22^ Other prospective longitudinal studies of healthy women in Mexico City, urban regions of Argentina, and rural Malawi reported GWG at term of 12.1 kg, 10.7 kg, and 3.7-6.4 kg,^49^ ^50^ ^51^ respectively, and large cross sectional studies of low risk Japanese women, well nourished women in Switzerland, and Swedish birth registry records have reported singleton term GWG of 10.0 kg, 15.5 kg, and 13.8 kg, respectively.^17^ ^47^ ^52^ All these studies were based on country specific populations and used various classifications of BMI status. Furthermore, most of them relied on recalled or routinely recorded weight measurements from medical records or weight data from large population databases with questionable measurement sources, validity, and reliability.

### Strengths and limitations of study

We recognise that our study has some limitations. As the first weight measurement was taken between 9 and 13^+6^ weeks’ gestation, the BMI classification of women as normal weight was not based on a value from before pregnancy. The results of the post hoc sensitivity analysis, however, were reassuring, and we believe that the effect of any possible misclassification is therefore small. Measurements before pregnancy are seldom available in clinical practice or research studies, especially in low risk women.^53^ ^54^ Recruitment of women who intend to conceive is also challenging and might be culturally unacceptable in some populations, which would introduce selection bias; this could explain why there are few studies with measured pre-pregnancy weight, which should ideally be used to construct GWG references or standards. Consequently, clinicians and researchers have often relied on self reported pre-pregnancy weight to estimate BMI and monitor GWG,^55^ despite the considerable limitations of error and recall bias.^56^ Another limitation is that it was not possible to infer the most appropriate GWG pattern for women who are underweight or obese as our population consisted only of healthy women with a BMI range of 18.5-<30. Underweight women are at increased risk for several adverse outcomes, including fetal growth restriction, so adequate GWG is especially important for this group.^5^ ^57^ ^58^ ^59^ Conversely, the growing problem of maternal obesity throughout the world has led to great interest in whether limiting GWG can reduce the risk of the associated adverse outcomes.^1^ It could be argued that the sample size is relatively small compared with epidemiological studies that have reported data from large populations—for example, the Danish National Birth Cohort of more than 60 000 women.^60^ It is always difficult to reach a balance between sample size and data quality, particularly when larger samples require the use of routinely collected clinical information. We decided when designing the study that it was more important to have a sufficiently large sample, collected prospectively in a scientifically robust manner, with standardised methods, quality control, and equipment, than a larger sample using data that have been routinely collected with less rigour and precision.

### Policy implications

Our results have several practical implications. Firstly, we are aware that in some settings, such as the UK, routine weight monitoring is not recommended.^15^ In most countries worldwide and in particular those with large populations at risk of under-nutrition, however, weight monitoring at antenatal visits is common practice. Our aim was to contribute to the standardisation of weight monitoring and the more systematic use of the data obtained. Overall, we suggest that the standards (as part of first level nutritional screening) can be used to alert clinicians to deviations in weight, triggering clinical inquiries as to whether such deviations are associated with complications related to pregnancy, medical conditions, or eating disorders. We would discourage clinicians, however, from telling women that deviations are due to pregnancy complications or recommending immediate behaviour changes as our data do not provide sufficient evidence for the standards to be interpreted in this way. Secondly, we believe that consideration should be given to referring women who are underweight before pregnancy for nutritional advice and treatment if necessary and that it is safe to suggest that during pregnancy such women should have GWG at least compatible with those of normal weight women. Finally, our data cannot be used to make recommendations to underweight, overweight, or obese women beyond those already provided by NICE.^61^

### Conclusions

In summary, we have described patterns of GWG among normal weight women that are compatible with desirable healthy pregnancy outcomes, which provide a basis to guide clinical recommendations on weight gain. To facilitate the use of such recommendations in clinical settings, epidemiological studies with data on important long term maternal and childhood outcomes are needed to identify optimal centile (that is, outcome based cut off points) categories associated with the best health outcomes. Towards that end, the INTERGROWTH-21^st^ Project is currently collecting one and two year follow-up data, including postpartum maternal weight patterns. We anticipate that the publication of this GWG standard will prompt debate among epidemiologists, nutritionists, obstetricians, and midwives about what the optimal thresholds should be. We believe that this standard is more robust than any other available charts and adds to the set of international standards from the INTERGROWTH-21^st^ Project, which aims to improve pregnancy care practices and outcomes by establishing benchmarks against which all women, their unborn babies, and newborns can be compared.^26^ ^27^ ^28^

**What is already known on this topic**

Guidelines and charts for gestational weight gain (GWG) that are currently in use around the world were derived from country specific studies
A recent systematic review assessing the quality of these studies has shown considerable heterogeneity in methods, in particular in terms of sample selection, study design, and methods of data collection and statistical analysis
This could explain the variation in recommendations and the lack of consensus regarding what constitutes adequate weight gain

**What this study adds**

This multi-country study of GWG adopted a prescriptive and highly standardised approach to describing the GWG patterns of normal weight women at low risk of adverse maternal and perinatal outcomes
The generated standards could be used to alert clinicians to deviations in weight, which should then initiate a series of questions to determine whether the changes are associated with complications related to pregnancy, medical conditions, or eating disorders
These standards are more scientifically robust than other published charts and add to the set of international standards from the INTERGROWTH-21^st^ Project

## Notes

We thank the health authorities in Pelotas, Brazil; Beijing, China; Nagpur, India; Turin, Italy; Nairobi, Kenya; Muscat, Oman; Oxford, UK; and Seattle, US, who facilitated the project by allowing participation of these study sites as collaborating centres. We are extremely grateful to Philips Medical Systems who provided the ultrasound equipment and technical assistance throughout the project. We also thank MedSciNet UK for setting up the INTERGROWTH-21^st^ website and for the development, maintenance, and support of the online data management system. We thank the parents and infants who participated in the studies and the more than 200 members of the research teams who made the implementation of this project possible. The participating hospitals included: Brazil, Pelotas (Hospital Miguel Piltcher, Hospital São Francisco de Paula, Santa Casa de Misericórdia de Pelotas, and Hospital Escola da Universidade Federal de Pelotas); China, Beijing (Beijing Obstetrics and Gynaecology Hospital, Shunyi Maternal and Child Health Centre, and Shunyi General Hospital); India, Nagpur (Ketkar Hospital, Avanti Institute of Cardiology Private Limited, Avantika Hospital, Gurukrupa Maternity Hospital, Mulik Hospital and Research Centre, Nandlok Hospital, Om Women’s Hospital, Renuka Hospital and Maternity Home, Saboo Hospital, Brajmonhan Taori Memorial Hospital, and Somani Nursing Home); Kenya, Nairobi (Aga Khan University Hospital, MP Shah Hospital and Avenue Hospital); Italy, Turin (Ospedale Infantile Regina Margherita Sant’ Anna and Azienda Ospedaliera Ordine Mauriziano); Oman, Muscat (Khoula Hospital, Royal Hospital, Wattayah Obstetrics and Gynaecology Poly Clinic, Wattayah Health Centre, Ruwi Health Centre, Al-Ghoubra Health Centre and Al-Khuwair Health Centre); UK, Oxford (John Radcliffe Hospital); and US, Seattle (University of Washington Hospital, Swedish Hospital, and Providence Everett Hospital). Full acknowledgment of all those who contributed to the development of the INTERGROWTH-21^st^ Project protocol appears at www.intergrowth21.org.uk **Members of the International Fetal and Newborn Growth Consortium for the 21^st^ Century (INTERGROWTH-21^st^) and its committees** **Scientific advisory committee **M Katz (chair from January 2011), MK Bhan, C Garza, S Zaidi, A Langer, PM Rothwell (from February 2011), D Weatherall (chair until December 2010). **Steering committee **ZA Bhutta (chair), J Villar (principal investigator), S Kennedy (project director), DG Altman, FC Barros, E Bertino, F Burton, M Carvalho, L Cheikh Ismail, WC Chumlea, MG Gravett, YA Jaffer, A Lambert, P Lumbiganon, JA Noble, RY Pang, AT Papageorghiou, M Purwar, J Rivera, CG Victora. **Executive committee **J Villar (chair), DG Altman, ZA Bhutta, L Cheikh Ismail, S Kennedy, A Lambert, JA Noble, AT Papageorghiou. **Project coordinating unit **J Villar (head), S Kennedy, L Cheikh Ismail, A Lambert, AT Papageorghiou, M Shorten, L Hoch (until May 2011), HE Knight (until August 2011), EO Ohuma (from September 2010), C Cosgrove (from July 2011), I Blakey (from March 2011), D Bishop (from February 2014). **Data analysis group **DG Altman (head), EO Ohuma, J Villar. **Data management group **DG Altman (head), F Roseman, N Kunnawar, SH Gu, JH Wang, MH Wu, M Domingues, P Gilli, L Juodvirsiene, L Hoch (until May 2011), N Musee (until June 2011), H Al-Jabri (until October 2010), S Waller (until June 2011), C Cosgrove (from July 2011), D Muninzwa (from October 2011), EO Ohuma (from September 2010), D Yellappan (from November 2010), A Carter (from July 2011), S Ash (from August 2011), D Reade (from June 2012), R Miller (from June 2012). **Ultrasound group **AT Papageorghiou (head), L Salomon (senior external advisor), A Leston, A Mitidieri, F Al-Aamri, W Paulsene, J Sande, WKS Al-Zadjali, C Batiuk, S Bornemeier, M Carvalho, M Dighe, P Gaglioti, N Jacinta, S Jaiswal, JA Noble, K Oas, M Oberto, E Olearo, MG Owende, J Shah, S Sohoni, T Todros, M Venkataraman, S Vinayak, L Wang, D Wilson, QQ Wu, S Zaidi, Y Zhang, P Chamberlain (until September 2012), D Danelon (until July 2010), I Sarris (until June 2010), J Dhami (until July 2011), C Ioannou (until February 2012), CL Knight (from October 2010), R Napolitano (from July 2011), S Wanyonyi (from May 2012), C Pace (from January 2011), V Mkrtychyan (from June 2012). **Anthropometry group **L Cheikh Ismail (head), WC Chumlea (senior external advisor), F Al-Habsi, ZA Bhutta, A Carter, M Alija, JM Jimenez-Bustos, J Kizidio, F Puglia, N Kunnawar, H Liu, S Lloyd, D Mota, R Ochieng, C Rossi, M Sanchez Luna, YJ Shen, HE Knight (until August 2011), DA Rocco (from June 2012), IO Frederick (from June 2012). **Neonatal group **ZA Bhutta (head), E Albernaz, M Batra, BA Bhat, E Bertino, P Di Nicola, F Giuliani, I Rovelli, K McCormick, R Ochieng, RY Pang, V Paul, V Rajan, A Wilkinson, A Varalda (from September 2012). **Environmental Health Group **B Eskenazi (head), LA Corra, H Dolk, J Golding, A Matijasevich, T de Wet, JJ Zhang, A Bradman, D Finkton, O Burnham, F Farhi. **Participating countries and local investigators** Brazil: FC Barros (principal investigator), M Domingues, S Fonseca, A Leston, A Mitidieri, D Mota, IK Sclowitz, MF da Silveira. China: RY Pang (principal investigator), YP He, Y Pan, YJ Shen, MH Wu, QQ Wu, JH Wang, Y Yuan, Y Zhang. India: M Purwar (principal investigator), A Choudhary, S Choudhary, S Deshmukh, D Dongaonkar, M Ketkar, V Khedikar, N Kunnawar, C Mahorkar, I Mulik, K Saboo, C Shembekar, A Singh, V Taori, K Tayade, A Somani. Italy: E Bertino (principal investigator), P Di Nicola, M Frigerio, G Gilli, P Gilli, M Giolito, F Giuliani, M Oberto, L Occhi, C Rossi, I Rovelli, F Signorile, T Todros. Kenya: W Stones and M Carvalho (co- principal investigators), J Kizidio, R Ochieng, J Shah,, S Vinayak, N Musee (until June 2011), C Kisiang’ani (until July 2011), D Muninzwa (from August 2011). Oman: YA Jaffer (principal investigator), J Al-Abri, J Al-Abduwani, FM Al-Habsi, H Al-Lawatiya, B Al-Rashidiya, WKS Al-Zadjali, FR Juangco, M Venkataraman, H Al-Jabri (until October 2010), D Yellappan (from November 2010). UK: S Kennedy (principal investigator), L Cheikh Ismail, AT Papageorghiou, F Roseman, A Lambert, EO Ohuma, S Lloyd, R Napolitano (from July 2011), C Ioannou (until February 2012), I Sarris (until June 2010). US: MG Gravett (principal investigator), C Batiuk, M Batra, S Bornemeier, M Dighe, K Oas, W Paulsene, D Wilson, IO Frederick, HF Andersen, SE Abbott, AA Carter, H Algren, DA Rocco, TK Sorensen, D Enquobahrie, S Waller (until June 2011).

Contributors: JV and SHK were responsible for conceiving the INTERGROWTH-21^st^ Project. JV, SHK, DGA, and JAN prepared the original protocol with later input from ATP, LCI, FCB, and ZAB. JV, ATP, LCI, AL, and ZAB supervised and coordinated the project’s overall undertaking. EOO performed the statistical analysis in collaboration with DGA. EOO, and DGA were responsible for data management and analysis in collaboration with JV. RP, FCB, WS, YAJ, MGG, and MP were collaborators and implemented the project in their respective countries. LCI, DCB, and EOO wrote the paper in collaboration with BFA and KR, with input from all co-authors. All co-authors read the report and made suggestions about its content. JV is guarantor.

Ethical approval: The INTERGROWTH-21^st^ Project was approved by the Oxfordshire research ethics committee ‘C’ (reference: 08/H0606/139), the research ethics committees of the individual participating institutions, and the corresponding regional health authorities where the project was implemented. All participants gave informed consent before taking part.

Transparency: The manuscript’s guarantor affirms that the manuscript is an honest, accurate, and transparent account of the study being reported; that no important aspects of the study have been omitted; and that any discrepancies from the study as planned (and, if relevant, registered) have been explained.

Data sharing: The relevant anonymised patient level data are available on reasonable request from the corresponding author.
